# Editorial: Update on epidemiology, endocrinology and treatment of cryptorchidism

**DOI:** 10.3389/fendo.2024.1435877

**Published:** 2024-06-10

**Authors:** Helena E. Virtanen, Katharina M. Main, Jorma Toppari

**Affiliations:** ^1^ Research Centre for Integrative Physiology and Pharmacology and Centre for Population Health Research, Institute of Biomedicine, University of Turku, Turku, Finland; ^2^ Department of Growth and Reproduction, Copenhagen University Hospital—Rigshospitalet, Copenhagen, Denmark; ^3^ Centre for Research and Research Training in Endocrine Disruption of Male Reproduction and Child Health (EDMaRC), Copenhagen University Hospital—Rigshospitalet, Copenhagen, Denmark; ^4^ Department of Clinical Medicine, Faculty of Health and Medical Sciences, University of Copenhagen, Copenhagen, Denmark; ^5^ Department of Pediatrics, Turku University Hospital, Turku, Finland; ^6^ InFLAMES Research Flagship Center, University of Turku, Turku, Finland

**Keywords:** testis, orchiopexy, maldescended testes, environment, endocrine disruptors, genetics

Cryptorchidism is the most common birth defect in boys and requires surgical treatment in ca. one percent of boys. Increased risk of fertility problems and testicular cancer are the long-term worries following cryptorchidism. Frontiers of Endocrinology is publishing a series of articles summarizing the current knowledge on this condition and future research needs. We here sum up some of the main messages of the reviews and the original genetic study.

Holmboe et al. reviewed the epidemiology of cryptorchidism and its potential risk factors, including endocrine disrupting chemicals. The incidence of cryptorchidism is 1–9% in term boys and cryptorchidism can also develop postnatally due to ascent of the testis. Our knowledge about the multifaceted etiology of congenital cryptorchidism has increased considerably over the last decades.

Population studies have shown geographical and ethnic differences in the prevalence of cryptorchidism as well as a temporal increase. Maternal lifestyle, i.e. smoking and alcohol consumption during pregnancy, use of mild analgesics, gestational diabetes, or fetal factors, i.e. intrauterine growth retardation or low birth weight, increase the risk of cryptorchidism.

The temporal increase of cryptorchidism prevalence, however, is thought to be related to external factors such as *in utero* exposure to endocrine disrupting chemicals (EDCs) from the environment. Evidence for this comes from observational ecological human studies, either performed as indirect exposure estimates from job matrices and geographic data or by longitudinal birth cohort studies measuring prenatal exposure to EDCs in maternal blood or urine, amniotic fluid, cord blood, placenta or breast milk in relation to birth outcome. EDCs comprise a very large number of non-persistent chemicals, i.e. phthalates, bisphenols, parabens, or persistent chemicals, i.e. dioxins, polychlorinated and polyfluorinated compounds, pesticides, and human exposure is continuous to many of these. Human studies have been corroborated by animal experiments where EDC exposure is strictly controlled for dosage and timing, and which also allow exposure to chemical mixtures relevant for the human scenario.

Genetic factors explain a small percentage of observed cases, often in familial cases or in cases related to more complex syndromes. Congenital defects in the Ras/MAPK genes cause RASopathies that underlie several syndromes. These include typical clinical features, one of which is often cryptorchidism. Juchnewitch et al. combined data from Estonia, Australia, Canada, and the USA in their original article on RASopathies in undiagnosed infertile men. Cryptorchidism in these men was associated with variants of classical Noonan syndrome genes *PTPN11*, *SOS1*, *SOS2*, or *LZTR1*.

As testis descent physiologically is first accomplished around gestational week 36, premature boys have a higher rate of undescended testes at birth which can spontaneously descend during the postnatal activation of the pituitary-gonadal axis. In addition, cryptorchidism is a risk factor for impaired spermatogenesis and subfertility as well as for testicular cancer in young adulthood. There is scientific evidence that early orchiopexy improves semen quality, whereas the benefit related to cancer risk is less clear.

Pakkasjärvi and Taskinen reviewed current concepts in surgical treatment of cryptorchidism and future directions. Several guidelines, such as those by European Association of Urologists, American Urological Association and Nordic guidelines recommend surgical treatment of congenital cryptorchidism between 6–12 months of age. Early treatment prevents germ cell damage and promotes adult reproductive health. Surgical options include both open and laparoscopic techniques, while the choice depends largely on the location and accessibility of the undescended testis.

Thorup et al. described the germ cell demise in cryptorcrhidism. Germ cell number declines rapidly in cryptorchidism during first years of life, partially attributed to physiologic apoptosis. Furthermore, germ cells fail to differentiate normally during mini-puberty leading to reduced germ cell proliferation and delayed clearance of gonocytes from the seminiferous epithelium. While proper temperature is an important factor in germ cell survival, it does not explain alone the risk of infertility and testicular cancer associated with cryptorchidism but there seems to be an underlying testicular pathology.

Only few studies have evaluated pubertal development in boys with a history of non-syndromic cryptorchidism. Rodprasert et al. reviewed such studies. It was found that timing of onset of puberty, physical development during puberty, and Leydig cell function are generally not affected among boys with a history of congenital cryptorchidism. However, the age at first conscious ejaculation may be delayed. Furthermore, especially a history of bilateral cryptorchidism treated with orchiopexy was associated with reduced testicular growth and increased FSH and decreased inhibin B levels during puberty, which suggests reduced Sertoli and/or germ cell number or function. These findings are in line with results from adult men with a history of surgically treated bilateral cryptorchidism. Findings also suggest more severe testicular pathology in those forms of cryptorchidism that needed orchiopexy than in spontaneously resolved forms. Studies focusing on pubertal development after acquired cryptorchidism are scarce and thus more studies are needed.

In their article, Rey and Grinspon reviewed the structure, expression and action of Anti-Müllerian hormone (AMH) secreted by immature testicular Sertoli cells. They also review the role of AMH in testicular descent, and its role as a biomarker of testicular function. During embryonic development, AMH provokes the regression of the Müllerian ducts. However, the role of AMH in fetal testicular descent is unknown. It may have a role in the INSL3-mediated swelling of the gubernaculum ligament during testicular descent. In boys with non-palpable gonads, serum AMH is a useful marker of existence of testicular tissue from the fetal stage until mid-puberty. In boys with palpable undescended testis, serum AMH reflects the functional immature Sertoli cell mass until midpuberty, when androgens cause maturation of the Sertoli cells and downregulate *AMH* expression in them. Measurement of AMH helps also in differential diagnostics of boys having cryptorchidism and associated genital anomalies.

These expert reviews give an excellent insight to etiology, pathology, and treatment of cryptorchidism. It is obvious that much more research is needed to understand the reasons for maldescent and how to prevent germ cell damage and testicular cancer.

## Author contributions

HV: Writing – review & editing, Writing – original draft. KM: Writing – review & editing, Writing – original draft. JT: Writing – review & editing, Writing – original draft.

